# Expanding the concepts of cancer metabolism

**DOI:** 10.1038/s12276-018-0070-9

**Published:** 2018-04-16

**Authors:** Sang-Min Jeon, Nissim Hay

**Affiliations:** 1 College of Pharmacy and Research Institute of Pharmaceutical Science and Technology (RIPST), Ajou University, Suwon, Gyeonggi-do 16499 Republic of Korea; 2 Department of Biochemistry and Molecular Genetics, College of Medicine, University of Illinois at Chicago, Chicago, IL 60607 USA

Since Otto Warburg first proposed that cancer cells have defective mitochondrial metabolism with enhanced aerobic glycolysis almost 100 years ago, uncovering the unique metabolic features of cancer has turned into one of the most vibrant and popular research areas in cancer research^1^. In contrast to Warburg’s idea of mitochondrial impairment, it is now evident that mitochondrial metabolism is largely intact, despite elevated glucose metabolism in cancer cells, and is even indispensable for tumor growth and survival^1–3^. Mitochondrial metabolic pathways, such as glutaminolysis, fatty acid oxidation, redox and one-carbon metabolism, are essential metabolic pathways for tumor growth and survival and thus are considered to be promising therapeutic targets for cancer therapy^4^. In addition, mitochondrial metabolism also confers resistance to conventional and targeted cancer therapy, implying that targeting mitochondrial metabolism could provide a promising combination strategy for cancer treatment^5^. Intriguingly, recent studies reveal that in addition to generating ATP, the mitochondrial electron transport chain is critical for tumor growth by providing an electron acceptor, such as NAD^+^, which is required for aspartate synthesis^6,7^. Importantly, it is now clear that the reprogramming of energy metabolism in cancer is directed by both intrinsic and extrinsic factors, such as oncogenic signaling and tumor microenvironment factors, which are actually known to cause cancer^8,9^.

In recent years, metabolomics has joined genomics and proteomics to advance our understanding of the complicated nature of cancer metabolism in vitro and in vivo^10,11^. In particular, ^13^C metabolic flux analysis (^13^C-MFA) has provided a major driving force to extend our understanding of metabolic alterations in cancer^12^. Integration of these technologies has enabled the discovery of complicated networks in glucose, amino acid and fatty acid metabolism in cancer^13^. Beyond the area of macronutrients, current research in cancer metabolism is expanding to micronutrients, such as vitamins, which were underexplored in a cancer metabolism context but are being revisited as emerging key players in cancer metabolism and are drawing great attention as promising therapeutic targets^14–17^. Further, beyond the canonical function of metabolic enzymes catalyzing nutrients, moonlighting functions of metabolic enzymes are also emerging as functional sensing mechanisms linking metabolic status to tumor growth and survival. A growing body of evidence suggests that some metabolic enzymes, such as hexokinase 2 and pyruvate kinase M2 (PKM2), may play a critical role in tumor growth and survival by not only their canonical metabolic activities but also their primarily non-enzymatic functions mediated through protein–protein interactions that regulate signal transduction or transcription^18,19^.

In this special issue of cancer metabolism, we invited four review articles highlighting the expanding features of cancer metabolism research, which include coverage of the technological aspects of cancer metabolism research with a practical guide for using ^13^C-MFA to a discussion of the emerging roles of new metabolic pathways involving vitamin D and non-metabolic functions of metabolic enzymes.

Gina DeNicola’s group (Moffitt Cancer Center and Research Institute) reviews recent advances in cancer metabolism from a technological perspective^20^. They elegantly summarize how diverse technologies, including chromatography-coupled mass spectrometry, metabolite tracing, seahorse, redox coupling, genome editing and integrated multi-omics, have contributed to deciphering and identifying the complicated network of metabolic pathways in cancer. Furthermore, they also provide practical information about the advantages and limitations of each analytical tool for metabolism analysis. Finally, they provide a comprehensive overview of how major findings concerning altered metabolism or metabolic pathways in cancer have been achieved to date.

Further, Maciek Antoniewicz (University of Delaware) provides an in-depth review of the technological aspects of cancer metabolism analysis, particularly focusing on ^13^C-MFA^21^. This review provides a practical guide for using ^13^C-MFA with a discussion on its advantages and potential pitfalls, which will be helpful for cancer biologists interested in analyzing and interpreting ^13^C-MFA data to study metabolism. Importantly, this tool has been indispensable for uncovering alterations in the metabolism of macronutrients, such as glucose, amino acids and fatty acids, in cancer.

As an example of a micronutrient important for cancer, Sang-Min Jeon’s group (Ajou University) reviews our current understanding of the metabolism and function of vitamin D in cancer^22^. Recently, vitamin D has been recognized as a key player in diverse physiological and pathological processes beyond bone and calcium metabolism. Moreover, accumulating epidemiological and experimental evidence suggest that vitamin D metabolism and function are critical for the prevention and treatment of a broad spectrum of cancers. This review provides a comprehensive summary of the metabolism of vitamin D and its hormonal regulation in physiology and proposed antitumorigenic functions. Moreover, the authors summarize the diverse mechanisms that lead to the dysregulation of vitamin D metabolism and its role in cancer, which contributes to the development of cancer and resistance to vitamin D-based cancer therapy. Finally, they also discuss future directions to improve the efficiency of vitamin D-based cancer therapy while minimizing its serious side effect of hypercalcemia.

Finally, Almut Schulze’s group (University of Wuerzburg) reviews the emerging non-canonical functions of key metabolic enzymes regulating signal transduction, transcription and the cytoskeleton^23^. Notably, they classify the non-canonical functions of metabolic enzymes into three categories with specific details: (1) enzymes with oncogenic non-canonical functions, such as protein kinases, transcription regulators or cytoskeleton modulators; (2) enzymes with tumor-suppressive non-canonical functions, such as counteracting hypoxia-inducible factor 1a and mitogen-activated protein kinase signaling or promoting p53 function; and (3) enzymes modulating non-canonical functions in a manner dependent on the metabolic state, such as integrating the metabolic state into the mitogenic signaling of mechanistic target of rapamycin complex 1, myelocytomatosis cellular oncogene or PKM2. They also discuss how future research is needed to better dissect the metabolic and non-metabolic functions of metabolic enzymes in cancer to design an effective cancer therapeutic strategy.

Beyond the role of aerobic glycolysis proposed by Warburg, our understanding of cancer metabolism has greatly evolved with the identification of new metabolic pathways using cutting-edge technologies. Now, cancer metabolism research is shifting into a new era with renewed hope for curing cancer and overcoming the limitations of conventional and targeted therapies^24,25^. Recently, the Food and Drug Administration approved a first-in-class cancer metabolism drug, enasidenib, which targets mutated isocitrate dehydrogenase 2 for acute myeloid leukemia, highlighting the promise of new drugs targeting metabolism^26^. In addition to enasidenib, multiple candidates targeting the Achilles’ heel of cancer metabolism are undergoing clinical trials^27^. Thus, it is likely that additional drugs targeting cancer metabolism will be developed for cancer therapy, either alone or in combination with conventional therapy, targeted therapy and immunotherapy.
